# High genetic heterogeneity of *Mycobacterium intracellulare* isolated from respiratory specimens

**DOI:** 10.1186/s12866-021-02426-5

**Published:** 2022-01-03

**Authors:** Nicoletta Lari, Laura Rindi

**Affiliations:** Dipartimento di Ricerca Traslazionale e delle Nuove Tecnologie in Medicina e Chirurgia, Università di Pisa, 35/39, I-56127 Pisa, Italy

**Keywords:** *Mycobacterium intracellulare*, Molecular typing, VNTR analysis

## Abstract

**Background:**

*M. intracellulare* is a frequent causative pathogen of nontuberculous mycobacteria infection that causes infections in the respiratory tract, whose incidence is increasing in many countries. This study aimed at determining the VNTR-based genetic diversity of a collection of 39 *M. intracellulare* human strains isolated from respiratory specimens over the last 5 years.

**Results:**

The VNTR analysis showed that *M. intracellulare* strains displayed a high genetic diversity, indicating that the *M. intracellulare* genotypes are quite heterogeneous in our geographical area. Moreover, a comparison with VNTR profiles of strains from other countries confirmed that genotypes of clinical strains of *M. intracellulare* are not related to geographical origin.

**Conclusions:**

VNTR typing has proved to be a highly discriminatory method for better understanding the molecular epidemiology of *M. intracellulare*.

## Background

*Mycobacterium intracellulare,* a member of *Mycobacterium avium* complex (MAC), is responsible for many of the human-associated nontuberculous mycobacteria pulmonary infections worldwide [1]. *M. intracellulare* is ubiquitous in the environment, including soil and water, and several studies reported drinking water or bathroom as sources of infection for human [2]. *M. intracellulare* is an important pathogen that causes infections in the respiratory tract in immunocompetent patients, showing more severe clinical characteristics and worse prognosis than infections caused by *M. avium* [3]. In Italy, as in many other countries worldwide, *M. intracellulare* is a frequent causative pathogen of nontuberculous mycobacteria infection and the incidence of *M. intracellulare* infections is increasing [4] . New strategies, based on the knowledge of epidemiology and biodiversity of clinical strains, aimed at the effective control of *M. intracellulare* infections in humans, are needed. The variable numbers of tandem repeats (VNTR) analysis is a rapid, highly reproducible and discriminatory genotyping method that has been successfully applied for MAC isolates, especially *M. avium* [5–8]. Recently, studies have been performed to select effective VNTR loci for *M. intracellulare* and to evaluate their epidemiological usefulness [9, 10].

In the present study, in light of the significant increase in *M. intracellulare* occurred in recent years, we determined the VNTR-based genetic diversity of a collection of *M. intracellulare* human strains isolated from 2015 to 2019 in order to determine molecular epidemiology and estimate the genetic relationships among *M. intracellulare* isolates in our setting.

## Results and discussion

**Fig. 1:**
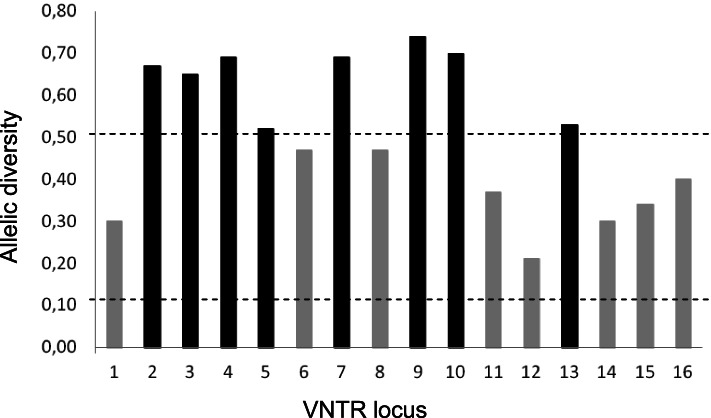
VNTR allelic distribution in 39 *M. intracellulare* clinical isolates. Black and grey bars indicate strains with high and medium diversity index, respectively

A total of 39 *M. intracellulare* human strains, isolated over a 5 year-period from the same number of TB patients resident in Tuscany, Italy, were genotyped by determining the polymorphism of a set of 16 VNTR loci [10]. Each of the 16 VNTR loci were efficiently amplified from all strains. First, the resolution provided by each VNTR locus was quantified by calculating its allelic diversity, which depends upon both the number and the distribution of the alleles, according to Selander et al. [11]. Table 1 shows the allelic diversity of the VNTR loci of our collection. Eight loci (VNTR-2, − 3, − 4, − 5, − 7, − 9, − 10 and − 13) had a high diversity index (*h* ≥ 0.5); eight loci (VNTR-1, − 6, − 8, − 11, − 12, − 14, − 15 and − 16) achieved a medium diversity index (0.1 ≤ *h* ≤ 0.5); none of the 16 loci showed a low diversity index (h ≤ 0.1) (Fig. 1). In agreement with previous reports [10, 12–14], the discriminatory power of VNTR typing yielded an HGDI of 0.993, confirming the great ability of the VNTR analysis to differentiate *M. intracellulare* isolates.

**Table 1:** VNTR allelic distribution in 39 *M. intracellulare* clinical isolates

VNTR locus	No. of isolates with the specified VNTR copy number											Allelic diversity
	0	1	2	3	4	5	6	7	8	9	10	
1	1	2	32	1	1	2						0.30
2	6	5	8	19				1				0.67
3	15	14	10									0.65
4		17	4	10	7			1				0.69
5	11	4	24									0.52
6	3	6	27	3								0.47
7	1	7	18	9					1		3	0.69
8	8	3	25	3								0.47
9	10	9	13	6	1							0.74
10	1	13	5		15	5						0.70
11		1	30	5	2	1						0.37
12		3	34	2								0.21
13	4		10	24		1						0.53
14	1	3	32	3								0.30
15		9	30									0.34
16	1	6	29	1	2							0.40

**Fig. 2:**
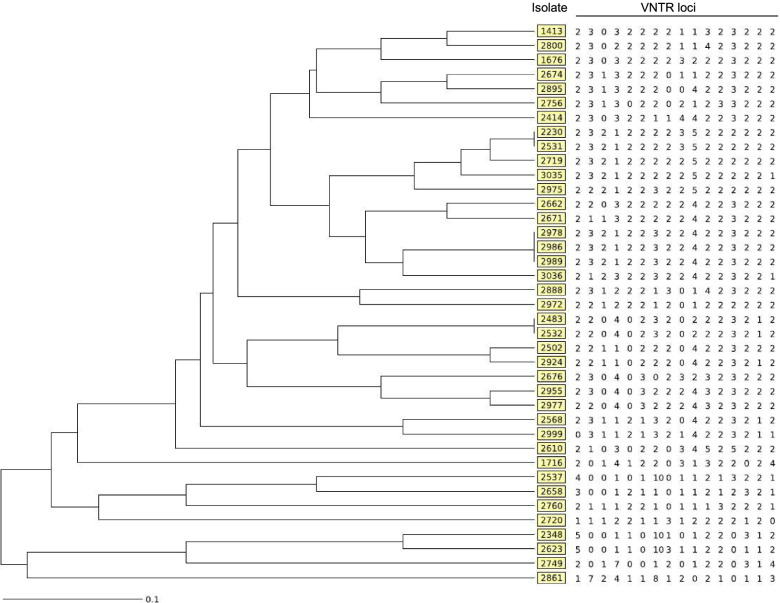
UPGMA dendrogram based on the allelic profiles of the 39 *M. intracellulare* strains

**Fig. 3:**
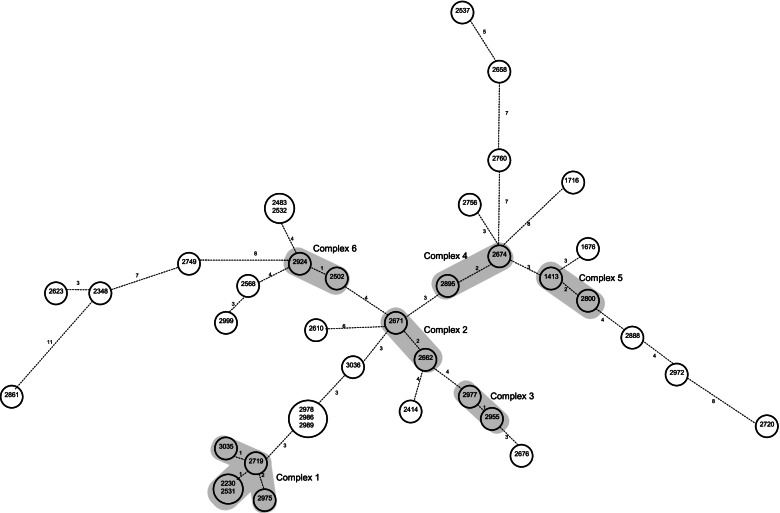
Minimum spanning tree of the 39 *M. intracellulare* strains. Each circle represents a VNTR profile; the number inside the circle identify the strain corresponding to the VNTR profile. Size of circles is proportional to the number of isolates sharing the VNTR profile. Numbers next to the branches indicate the level of changes induced by loss or gain of VNTR copies at a given locus, yielding a change from one allele to another. Surrounding grey indicates the clonal complexes

The VNTR analysis was used to construct a dendrogram, reported in Fig. 2, in which the VNTR patterns were ordered by similarity. Our VNTR analysis revealed 35 distinct VNTR genotypes; of these, 32 patterns were unique, while 3 patterns were shared by multiple isolates, thus yielding 2 clusters consisting of 2 strains and 1 cluster of 3 strains. The genetic relationships between the study isolates were also assessed by construction of a minimum spanning tree (Fig. 3). The minimum spanning tree, based on variations from one allele to another due to the loss or gain of one sequence at a single VNTR locus, allowed us to distinguish six clonal complexes. Complex 1 included 5 isolates, 2 of which were grouped in a cluster. The other five complexes were composed of 2 isolates with unique VNTR profile.

On the whole, the results obtained through the VNTR analysis showed that *M. intracellulare* strains displayed a high genetic diversity, indicating that the *M. intracellulare* genotypes are quite heterogeneous in our geographical area. These data differs to what has recently been demonstrated for *M. avium* subsp. *hominissuis* strains, isolated in the same geographical area, that displayed a close genetic relationship [15], and therefore support the hypothesis that *M. intracellulare* and *M. avium* may have different sources of infection and transmission pathways, as suggested in a recent studies [6, 14]. However, this study has some limitations: the population size was small and phenotypic features such as drug susceptibility and clinical characteristics of the strains were not investigated.

**Fig. 4:**
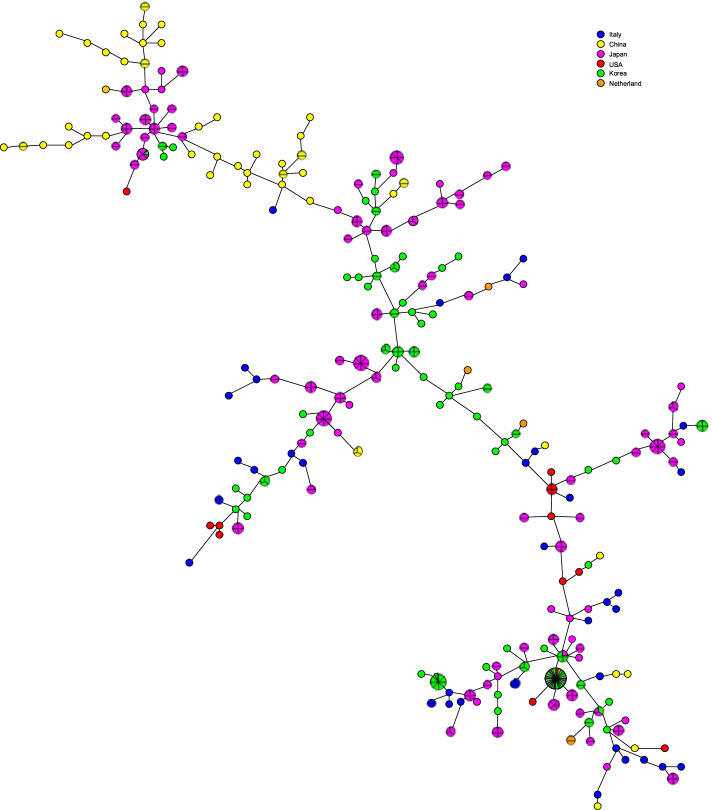
Minimum spanning tree of 454 *M. intracellulare* strains. The genetic relationships between 39 isolates from Italy (this study), 226 from Japan [6, 10, 16], 115 from Korea [6, 17], 52 from China [13], 15 from United States [6] and 7 from The Netherlands [6] were visualized. Each circle represents a VNTR profile; size of circles is proportional to the number of isolates sharing the VNTR profile. The length of the lines connecting isolates are not proportional to the number of allelic variation between the isolates

Moreover, in order to evaluate the impact of geographical origin on *M. intracellulare* genotypes, our 16-loci VNTR results were compared with data of clinical isolates from other countries. In this regard, in addition to the 39 *M. intracellulare* strains characterized in this study, 226 *M. intracellulare* clinical isolates from Japan [6, 10, 16], 115 from Korea [6, 17], 52 from China [13], 15 from United States [6] and 7 from the Netherlands [6] were considered. As shown in Fig. 4, representing the minimum spanning tree obtained by comparing a total of 454 *M. intracellulare* clinical strains, isolates from different countries were distributed overall, indicating that no correlation between the 16-loci VNTR genotypes and the geographical region of strains isolation was observed. This comparative analysis integrates and confirms findings of other studies demonstrating that genetic characteristics of clinical strains of *M. intracellulare* are not related to geographical origin [6, 17]. On the other hand, the VNTR analysis, which has proved to be a very useful and highly discriminatory tool in molecular epidemiology studies, is unable to recognize species related to *M. intracellulare*, such as *Mycobacterium paraintracellulare* and *Mycobacterium indicus pranii*. The identification of clinical isolates genetically close to *M. intracellulare*, addressed by multigene sequence-based analysis [18] or comparative genomic analysis [19], is important to define virulence determinants and evolution of MAC strains causing pulmonary disease.

## Conclusions

The evaluation of the genetic diversity of *M. intracellulare* strains isolated from respiratory specimens over a 5-year period, carried out using the 16-loci VNTR analysis, provided the identification of circulating genotypes in a region of Italy. Our study showed a high genetic heterogeneity of *M. intracellulare* isolates and confirmed that genotypes of clinical strains of *M. intracellulare* are not related to geographical origin. VNTR typing has proved to be a highly discriminatory method for better understanding the molecular epidemiology of *M. intracellulare*.

## Methods

### Clinical isolates

A total of 39 *M. intracellulare* strains, identified by GenoType NTM-DR test (Hain Lifescience), able to discriminate between *M. intracellulare* and *Mycobacterium chimaera*, were isolated between 2015 and 2019 from the same number of patients (23 males and 16 females; 70% older than 65 years) resident in Tuscany, Italy, with pulmonary infections at the Laboratory of Clinical Mycobacteriology of the University Hospital of Pisa, Italy.

### VNTR analysis

Genomic DNA was extracted by the cetyltrimethyl-ammonium bromide (CTAB) method. VNTR typing was performed by PCR using specific primers for 16 *M. intracellulare* VNTR loci, as described previously [10]. The PCR fragments were analyzed by gel electrophoresis using 2% NuSieve agarose (Cambrex Bio Science Rockland). For each locus, sizes of amplicons were estimated by comparison with 20 bp and 100 bp markers (Superladder-low; GenSura, CA, USA. VNTR profile is expressed as a string of 16 numbers, each representing the number of tandem repeats (TR) at a given VNTR position.

### VNTR allelic diversity and genetic relationships analysis

The allelic diversity of the VNTR loci was calculated according to Selander’s formula [11]. The global discriminatory power of complete VNTR scheme was determined using the Hunter and Gaston discriminatory index (HGDI) [20].

VNTR data were analyzed by the MIRU-VNTR*plus* web application available at www.miru-vntrplus.org; VNTR profile similarities were visualized by generating a dendrogram using the unweighted pair group method with arithmetic averages (UPGMA); the genetic relationships among the isolates were analyzed by constructing a minimum spanning tree, an undirected network in which all the VNTR profiles are linked together with the smallest possible linkages between nearest neighbours, by the UPGMA method.

## Data Availability

All data generated or analysed during this study are included in this published article.
